# Profiling of Secondary Metabolites of Optimized Ripe Ajwa Date Pulp (*Phoenix dactylifera* L.) Using Response Surface Methodology and Artificial Neural Network

**DOI:** 10.3390/ph16020319

**Published:** 2023-02-20

**Authors:** Fanar Alshammari, Md Badrul Alam, Marufa Naznin, Ahsan Javed, Sunghwan Kim, Sang-Han Lee

**Affiliations:** 1 Department of Food Science and Biotechnology, Graduate School, Kyungpook National University, Daegu 41566, Republic of Korea; 2 Food and Bio-Industry Research Institute, Inner Beauty/Antiaging Center, Kyungpook National University, Daegu 41566, Republic of Korea; 3 Department of Chemistry, Kyungpook National University, Daegu 41566, Republic of Korea; 4 Mass Spectrometry Converging Research Center and Green-Nano Materials Research Center, Daegu 41566, Republic of Korea

**Keywords:** Ajwa dates, antioxidant, artificial neural network, polyphenolics, response surface methodology

## Abstract

The date palm (*Phoenix dactylifera* L.) is a popular edible fruit consumed all over the world and thought to cure several chronic diseases and afflictions. The profiling of the secondary metabolites of optimized ripe Ajwa date pulp (RADP) extracts is scarce. The aim of this study was to optimize the heat extraction (HE) of ripe Ajwa date pulp using response surface methodology (RSM) and artificial neural network (ANN) modeling to increase its polyphenolic content and antioxidant activity. A central composite design was used to optimize HE to achieve the maximum polyphenolic compounds and antioxidant activity of target responses as a function of ethanol concentration, extraction time, and extraction temperature. From RSM estimates, 75.00% ethanol and 3.7 h (extraction time), and 67 °C (extraction temperature) were the optimum conditions for generating total phenolic content (4.49 ± 1.02 mgGAE/g), total flavonoid content (3.31 ± 0.65 mgCAE/g), 2,2-diphenyl-1-picrylhydrazyl (11.10 ± 0.78 % of inhibition), and cupric-reducing antioxidant capacity (1.43 µM ascorbic acid equivalent). The good performance of the ANN was validated using statistical metrics. Seventy-one secondary metabolites, including thirteen new bioactive chemicals (hebitol II, 1,2-di-(syringoyl)-hexoside, naringin dihydrochalcone, erythron-guaiacylglycerol-β-syringaresinol ether hexoside, erythron-1-(4′-*O*-hexoside-3,5-dimethoxyphenyl)-2-syrngaresinoxyl-propane-1,3-diol, 2-deoxy-2,3-dehydro-N-acetyl-neuraminic acid, linustatin and 1-deoxynojirimycin galactoside), were detected using high-resolution mass spectroscopy. The results revealed a significant concentration of phytoconstituents, making it an excellent contender for the pharmaceutical and food industries.

## 1. Introduction

Extraction is the first and most significant step in recovering and purifying bioactive chemicals from plant sources, and it is often accomplished using maceration, distillation, or Soxhlet reflux extraction, but long extraction times and low extraction effectiveness limit these approaches [1]. For large-scale extraction, the best approach should provide high efficiency with minimal processing time. In general, either empirical or statistical methods can be used to optimize a process [2,3]. The one-factor-at-a-time technique is an empirical system that involves changing one element at a time while maintaining all other variables constant [4]. The main limitation of this strategy is that it neglects the interplay between the factors, and hence, it cannot account for all the effects of a parameter on the response. One more burden is that it requires numerous trials to finish the study, thereby increasing the time, costs, and reagent and material consumption [5]. To solve this challenge, multivariate statistical approaches were adopted for optimizing analytical procedures. One of the most prominent multivariate strategies used in analytical optimization is the response surface methodology (RSM) [4]. RSM is a collection of statistical and mathematical methodologies for constructing, developing, and adjusting procedures in which many variables impact a desired response, with the purpose of optimizing that response. It can be used to develop, formulate, and create new products, as well as refining the design of current ones. It describes how the independent variables influence the processes, either singly or together. This experimental approach provides a mathematical model that illustrates the chemical or biological procedures in addition to assessing the impact of independent factors [5,6]. However, any nonlinear relationship between the variables may reduce the forecast accuracy of RSM [7]. Artificial neural networks (ANNs) are rapidly being utilized as prediction tools in a variety of disciplines, including engineering, owing to their capacity to use learning algorithms to identify input–output links for nonlinear complex systems [8].

The date palm (*Phoenix dactylifera* L., Arecaceae family) is a popular edible fruit consumed all over the world. The Ajwa date is only cultivated in Medina, Saudi Arabia. It is one of the most expensive and valued cultivars on the market owing to religious and ethnomedical beliefs regarding its health-promoting qualities [9]. The beneficial properties of Ajwa dates are mentioned in the Old Testament, “Hadith”, and Islamic literature, and eating these dates is thought to cure several chronic diseases and afflictions [9]. It is regarded to have cardioprotective [10], hepatoprotective [11], nephroprotective [12], and constipation-relieving [13] properties and antioxidant, anti-inflammatory [14], anticancer [15], antifungal, antibacterial, and antiviral activities [16]. The fruit is rich in dietary fiber, minerals, organic acids, and vitamins and has great nutritional and therapeutic value [13]. Carbohydrates constitute more than 70% of the fruit [17]. In addition, it contains abundant bioactive components such as polyphenols, including phenolic acids, flavonoids, and lignans [13].

The optimization of the extraction of various dates from Tunisia, Algeria, Egypt, and other locations and their polyphenolic content as well as antioxidant activities were described in the prior literature; however, no systematic statistical technique was applied [1,18,19]. Additionally, the majority of the optimization of the extraction process was performed solely using RSM methodology, but the illustrious scientists made no attempt to compare the efficacy of predictive modeling with alternative, more effective methods such as ANN. Furthermore, to the best of our knowledge, this is the first study to use heat extraction (HE) with RSM and ANN to enhance the polyphenolic components and antioxidant activity of ripe Ajwa date pulp (RADP) extracts. The aim was to use the RSM central composite design (CCD) tool to investigate and optimize extraction parameters such as extraction temperature, time, and ethanol concentration to acquire the maximum polyphenolic content and antioxidant potentiality from RADP. We argue that the projected values generated by the RSM-CCD approach correspond to the actual results and that this statistical tool is an effective model to optimize RADP polyphenolic compound extraction and antioxidant activity. The estimating capabilities and modeling effectiveness of the RSM and ANN models were also statistically examined. Additionally, we have also profiled the secondary metabolites of RADP using high-resolution mass spectrometry analysis.

## 2. Results and Discussion

### 2.1. Fitting of the RSM and ANN Models

Table 1 summarizes the experimental parameters and findings of 20 extraction situations. All response variables were transformed into second-order quadratic polynomial equations to account for the difference in the various replies as functions of the extraction factors. The statistical significance of the fitted second-order quadratic model equations were determined using ANOVA (Table 2). To improve the model fit and prediction, non-significant terms (*p* > 0.05) were removed from the models. The *p*-values were used to determine the importance of each coefficient. The model terms were considered significant, very significant, and strikingly significant when the *p*-values were less than 0.05, 0.01, and 0.001, respectively; the model terms were not significant when the *p*-values were greater than 0.05 [20].

**Figure 1:**
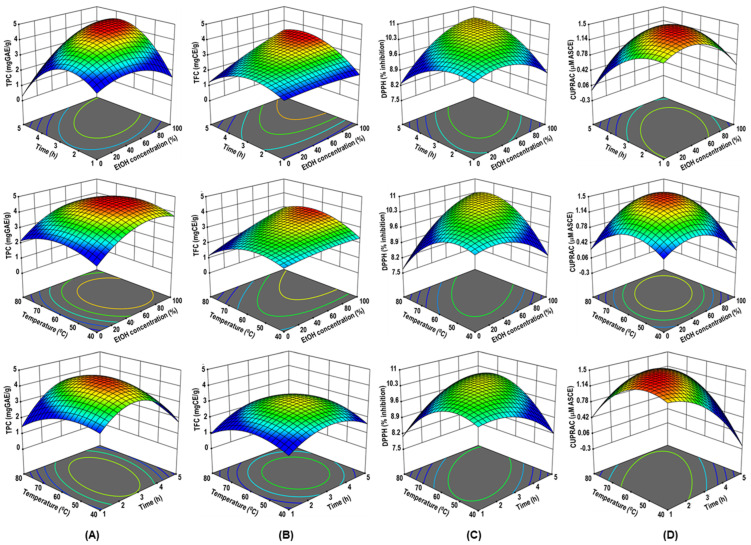
The three-dimensional (3D) response surface plots of RADP extraction on ethanol concentration, time, and temperature for TPC (**A**), TFC (**B**), DPPH radical scavenging activity (**C**), and CUPRAC (**D**) as a function of significant interaction factors for RSM.

The statistical test known as the F-value, which compares the source mean square to the residual square, is frequently employed in conjunction with the *p*-value. The better the F-value, the more significant the model [21]. Table 2 outlines the model F-value range, which runs from 34.85 to 154.89. Adjusted R^2^ adjusts for the number of terms in the model, whereas projected R^2^ measures the variation in new data explained by the model. The various R^2^ values represent the degree of interpretation concerning the mean that the model can explain. For a fair level of agreement, the maximum expected difference between adjusted R^2^ and predicted R^2^ is 0.2; otherwise, it may indicate a problem with the model or the experimental data used to create the model [22]. The model is more accurate if adjusted R^2^ and projected R^2^ values are near 1. The constructed regression models, therefore, have a high level of statistical significance, as shown by their R^2^ values (0.9691–0.9929), adjusted R^2^ value (0.9413–0.9865), and predicted R^2^ value (0.8432–0.9580). It is important to note that R^2^ is ineffective for establishing the effectiveness or validity of a nonlinear model [23]. Contrarily, the most widely used metrics among the numerous model selection techniques are the predicted residual sum of squares (PRESS), the Akaike information criterion (AIC), and the Bayesian information criterion (BIC) [24]. The PRESS measures a model’s propensity to predict; hence, the lower the PRESS score, the greater the model’s prediction [25]. The PRESS value range in the current study is between 0.0464 and 0.5369, which supports the claim. Furthermore, it is generally known that Schwarz’s Bayesian information criterion (BIC) and Akaike information criterion (AIC) are both penalized-likelihood information criteria. In contrast to BIC, which is an estimate of the posterior probability of a model being authentic in a specific Bayesian setup, AIC is an estimate of a constant plus the distance between the fitted likelihood function of the model and the unknown actual likelihood function of the data [26]. The lower AICc and BIC model is typically considered the “best” model. AICc and BIC values, in this case, vary from −55.62 to −4.85 and −70.11 to −19.33, respectively, and indicate that the intended responses can be predicted with great accuracy. Furthermore, the appropriate precision value is an indicator of the signal-to-noise ratio. It is preferable to have a ratio >4 [27]. Here, the ratio was between 18.9896 and 34.9882, suggesting a sufficient signal, indicating that the model is suitable for this procedure. The coefficient of variation (CV) is a measure of a model’s reproducibility; in general, if CV < 10%, the model is reproducible [28]. In this study the CV values range from 0.9981 to 3.44. The modified R^2^ (R^2^ ≥ 0.80) was well within acceptable limits in this study, showing that the experimental data fits second-order polynomial equations satisfactorily [29]. To demonstrate the interactions between the independent variables, 3D surfaces and contour plots were constructed using multiple linear regression equations. The main and cross-product effects of the independent variables on the response variables are more easily understood from these 3D charts (Figure 1A–D).

There is growing body of evidence that ANN modeling is superior and more sophisticated than RSM and that ANNs are an emerging viable alternative to the RSM system for complicated nonlinear multivariate modeling. In terms of fitting experimental responses, prediction, and modeling of biological processes, ANNs are more precise than RSM [30,31]. The experimental values were subjected to ANN modeling for further model verification. The predicted values obtained after training the ANN model are given in supplementary data Table S1.

The ANN model predicted values that were reasonably close to the observed values, indicating the appropriateness of our model. The nonlinear correlations between the extraction parameters (X_1_, X_2_, and X_3_) and response variables (Y_1_, Y_2_, Y_3_, and Y_4_) were predicted using the ANN model. By comparing the error between network training and testing, the number of neurons in the hidden layer was modified using the hit-and-trial strategy. During development, the input layer was not triggered using the transfer function, but the output and hidden layers were activated using pure line (purelin) and tangent sigmoid transfer (tansig) functions in MATLAB. In the experiment, the lowest feasible error between training and testing was evaluated, and the minimum number of epochs to prevent model overfitting was considered; the results were consistent with the findings of previous works [32]. Training the network with the Levenberg–Marquardt technique yielded the best validation performance for all dependent variables: TPC (Y_1_), TFC (Y_2_), DPPH (Y_3_), and CUPRAC (Y_4_) (supplementary data Figures S1–S4).

### 2.2. Comparison of the Prediction Abilities of the RSM and ANN Models

The predictive and estimate capabilities of RSM and ANN models were compared. Comparative similarity plots were used to examine the projected values of the four target responses (Y_1_, Y_2_, Y_3_, and Y_4_) obtained using the ANN model. The results indicate that compared to the RSM model, the ANN model was more correct, precise, and capable of assessment in terms of fitting the experimental data to all target responses (supplementary data Table S1). The RSM model had a greater degree of divergence between the projected and actual data than the ANN model, whereas the ANN model had stable residuals with low variation.

The values of the correlation coefficient (R^2^), root-mean-square error (RMSE), absolute average deviation (AAD), and standard error of prediction (SEP) were calculated to compare the RSM and ANN models (Table 3). Better modeling requires lower RMSE, AAD, and SEP, and greater R^2^. The calculated R^2^ values of the trained ANN model were higher than those of the RSM model, indicating the superiority of the ANN model in terms of predicting all four dependent variables. The AAD is a measure of the projected data’s divergence from the actual data, whereas the RMSE numbers demonstrate the model’s absolute fit. The ANN model performed better than the RSM model, as seen by the former’s lower AAD and RMSE values. In addition, the ANN model had low SEP values, in the range of 0.0449–0.2903. The ANN model has a substantially stronger predictive capacity than the RSM model because the former can approximate nonlinear systems, but the latter is only successful if the system is constrained to second-order polynomial regression. The ANN model is likewise unaffected by experimental design, and it is faster at calculating many responses in a single run than the RSM model, which requires multiple runs for multi-response optimization in a typical experimental design [33]. Unlike the RSM model, building an ANN model requires many iterative calculations and long computation time. Moreover, ANNs are also useful as they are flexible for the addition of new experimental data for model generation [33].

### 2.3. Effect of HE Parameters on the TPC and TFC

The TPC and TFC in the RADP extracts varied from 2.87 to 4.53 mgGAE/g and 2.03 to 3.44 mgCAE/g, respectively (Table 1). The linear effect of ethanol concentration (*X_1_*), temperature (*X*_3_), and the quadratic component of (*X*_1_^2^), (*X*_2_^2^), and (*X*_3_^2^) as well as the interaction of (*X*_1_*X*_3_) and (*X*_2_*X*_3_) were significant for TPC. The linear effect of (*X*_1_), (*X*_2_), and (*X*_3_); the quadratic component of (*X*_2_^2^) and (*X*_3_^2^); and the interaction of (*X*_1_*X*_2_) and (*X*_1_*X*_3_) were significant for TFC (Table 2). The following second-order polynomial equations shown in Equations (1) and (2) demonstrate the relationships among TPC, TFC, and their variables.

(1) $$\begin{array}{c} TPC\left(Y_{1}\right)=4.45+0.3400X_{1}+0.0200X_{2}-0.0575X_{3}-0.2089X_{1}^{2}-0.4001X_{2}^{2}-0.1676X_{3}^{2}+0.2700X_{1}X_{2}\\ \;\;\;\;\;\;\;-0.0050X_{1}X_{3}+0.1425X_{2}X_{3} \end{array}$$

(2) $$\begin{array}{c} TFC\left(Y_{2}\right)=3.05+0.2388X_{1}+0.1113X_{2}-0.0525X_{3}-0.0328X_{1}^{2}-0.2116X_{2}^{2}-0.1853X_{3}^{2}+0.1450X_{1}X_{2}\\ \;\;\;\;\;\;\;+0.1050X_{1}X_{3}+0.0475X_{2}X_{3} \end{array}$$

The lack of fit values for TPC and TFC (F = 1.73 and 2.07, respectively) were non-significant, indicating that the model accurately predicted R^2^ = 0.9866 (TPC) and 0.9729 (TFC) and Adj.R^2^ = 0.9745 (TPC) and 0.9485 (TFC) (Table 2). The RSM model adequately predicted the effects of parameters on the TPC and TFC of the RADP extract. As shown in Figure 1A,B, when the temperature (*X*_3_) was fixed (60 °C), the maximum TPC and TFC were achieved when the ethanol concentration (*X*_1_) was 50%, and time was 3 h. This could be because a medium concentration of ethanol may make the solvent more polar and dissolve more polyphenols, both polar and moderately polar ones [1]. Experiments in a previous comparative study revealed that the extraction of polyphenols from green tea leaves using a high hydrostatic pressure procedure augmented as the percentage of ethanol in the solvent, which peaked at 50% ethanol and dropped after that [34]. Hence, extraction of polyphenols in hydroalcoholic solution is highly efficient as the polyphenols are highly soluble in these solutions [1]. Furthermore, when ethanol is present at a moderate quantity in water, it can disrupt and break the architecture and structure of phospholipids that make up the lipid bilayer of membranes, affecting the penetrability of plant cells, thereby allowing better extraction and diffusion of the polyphenolic compounds [34].

### 2.4. Effect of HE Parameters on the In Vitro Antioxidant Capacity (AC)

DPPH radical scavenging activity and CUPRAC analysis revealed a linear significant effect of ethanol concentration (*X*_1_) and the quadratic effect of temperature (*X*_3_) on antioxidant activity. The fitted second-order polynomial equations for DPPH (% inhibition) and CUPRAC (ascorbic acid equivalent μM) are shown in Equations (3) and (4):

(3) $$\begin{array}{c} DPPH\left(Y_{3}\right)=10.39+0.1706X_{1}+0.0444X_{2}-0.0069X_{3}-0.1399X_{1}^{2}-0.1374X_{2}^{2}-0.2124X_{3}^{2}+0.1837X_{1}X_{2}\\ \;\;\;\;\;\;+0.2437X_{1}X_{3}+0.2112X_{2}X_{3} \end{array}$$

(4) $$\begin{array}{c} CUPRAC\left(Y_{4}\right)=1.43+0.0419X_{1}-0.1144X_{2}+0.0481X_{3}-0.1074X_{1}^{2}-0.0886X_{2}^{2}-0.1086X_{3}^{2}\\ \;\;\;\;\;\;+0.0963X_{1}X_{2}+0.0762X_{1}X_{3}+0.1363X_{2}X_{3} \end{array}$$

The AC values ranged from 9.36% to 10.81% inhibition of DPPH and from 0.75 to 1.47 μM ascorbic acid equivalent (Table 1). The ANOVA results show that the data fitted the model results for DPPH (R^2^ = 0.9691 and Adj. R^2^ = 0.9413) and CUPRAC response (R^2^ = 0.9929 and Adj. R^2^ = 0.9865), and the lack of fit was non-significant (F = 1.03 for DPPH and 2.37 for CUPRAC) (Table 2). From the 3D response surface plots shown in Figure 1C,D, both DPPH radical scavenging activity and CUPRAC activity increase with increasing ethanol concentration to reach the maximum values at 100% and 75% ethanol, respectively. This means that the greater the quantity of the organic solvent, the better the electron and proton donation capacities are. This result is consistent with the prior finding for TFC that 100% ethanol is required for maximum extraction [34]. Increasing the ethanol concentration allows for the extraction of significant polyphenolics in RADP, both in terms of quality and quantity. There is increasing evidence that the quality and quantity of polyphenolic compounds and antioxidant activity differ with the ethanol concentrations [35,36].

### 2.5. Model Validation

**Figure 2:**
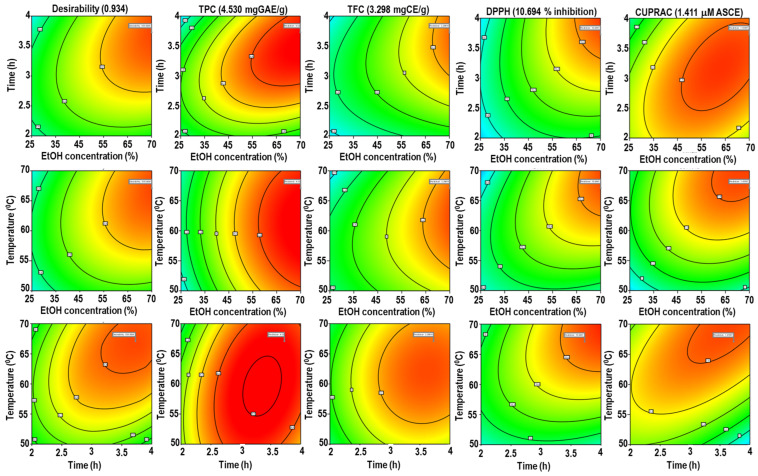
Desirability surface plot as a function of ethanol concentration, extraction time, and temperature for TPC, TFC, DPPH, and CUPRAC.

The desirability function optimizes responses for polyphenolic content (TPC and TFC) and antioxidant activity (DPPH and CUPRAC) concurrently. The parameters were forecasted using Derringer’s desirability function, allowing a multivariate analysis to discover the ideal level for all responses in a single extraction [37]. In this study, the following conditions were used to achieve the maximal overall desirability D = 0.934 (on a scale of 0 to 1): X_1_, 75%; X_2_, 3.7 h; and X_3_, 67 °C. Figure 2 shows the contour plot as a function of ethanol concentration, extraction time, and temperature at optimum condition. To verify the sufficiency of the model equations, a duplicate experiment was conducted in the optimal conditions predicted by Derringer’s desire model. The following results were obtained: TPC = 4.49 ± 1.02 mgGAE/g, TFC = 3.31 ± 0.65 mgCAE/g, % inhibition of DPPH = 11.10% ± 0.78%, and µM ascorbic acid equivalent CUPRAC = 1.43 ± 0.43. As stated in Table 4, the relative standard deviations (RSDs) of TPC, TFC, DPPH, and CUPRAC showed that the predicted values for all groups were very similar to the experimental results. This result is also supported by prior research [29].

### 2.6. Identification of Secondary Metabolites in RADP with High-Resolution Mass Spectrometry

Secondary metabolites in the RADP extracts were identified using ESI-MS/MS in the positive and negative ionization modes. As indicated in Table 5, 71 compounds were identified in the negative mode using MS^n^ data from the mass of the precursor ion, fragments, recognized fragmentation patterns for the given classes of compounds, and neutral mass loss, and from comparison with the existing literature and searches in online databases. Furthermore, the significance of these results was determined by finding the confidence level. Level 3 denotes a tentative candidate, whereas level 2 indicates the probable structure of the identified compound [38].

#### 2.6.1. Phenolic Acids

**Figure 3:**
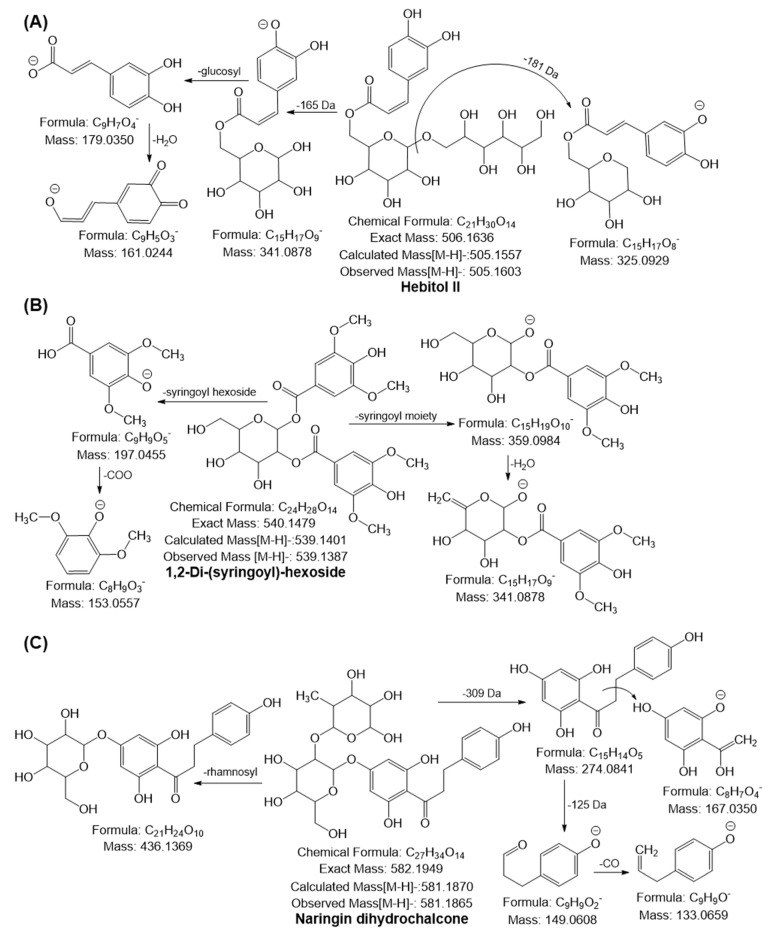
Possible mass fragmentation behavior of identified compounds in RADP. (**A**) Hebitol II, (**B**) 1,2-di-(syringoyl)-hexoside, and (**C**) naringin dihydrochalcone.

A phenolic acid may lose its methyl (15 Da), hydroxyl (18 Da), or carboxyl (44 Da) moiety to form a specific fragment ion. The fragmentation of a phenolic acid glycoside begins with the cleavage of the glycosidic link to yield the *m*/*z* of the phenolic acid and the corresponding loss of the sugar molecule (neutral mass loss of 162 Da). Thus, compounds **2**–**7** were tentatively identified as hydroxybenzoylhexose, coumaroyl hexose, caffeoylshikimic acid, caffeic acid hexoside, caffeic acid derivatives, and dicaffeoyl shikimic acid, respectively [17,39,40]. Among these, coumaroyl hexose (IC_50_ value: 37.7 μM), caffeoylshikimic acid (IC_50_ value: 2.9 μM), caffeic acid hexoside (IC_50_ value: 0.55 μM), and dicaffeoyl shikimic acid (IC_50_ value: 0.38 μM) were found to have DPPH-scavenging activity [41,42,43]. Additionally, coumaroyl glucoside severely inhibits glycogen phosphorylase, a crucial target for producing antihyperglycemic medicines [44]. The formation of advanced glycation end products (AGEs) and the activity of the angiotensin-converting and acetylcholinesterase enzymes are all inhibited by caffeine acid glucoside [45]. In addition, compound **1** yielded a precursor ion [M–H]^−^ at *m*/*z* 139.0050, generated characteristic ions at *m*/*z* 111.01 and 95.01 by loss of neutral molecules of CO and COO, respectively, and was provisionally identified as coumalic acid; this is the first time that this compound was identified in Ajwa dates (supplementary data Figure S5A). Furthermore, compound **8** produced a monoisotopic ion [M–H]^−^ at *m*/*z* 505.1603 and yielded fragment ions at *m*/*z* 325.09 [M-H-181 Da], *m*/*z* 341.08 [M-H-165 Da], and MS^2^ ion at *m*/*z* 341.08. Further loss of glucosyl moiety (162 Da) led to the generation of an ion at *m*/*z* 179.03, and the following loss of water molecules yielded an ion at *m*/*z* 161.02, thereby confirming the presence of caffeoyl hexoside. Based on the fragmentation behavior, compound **8** was tentatively confirmed as hebitol II, which has been identified in RADP for the first time (Figure 3A). According to Wang et al., hebitol II prevented the growth of *Plasmodium falciparum* 3D7, a malarial parasite line [46]. Moreover, compound **9** was tentatively identified as 1,2-di-(syringoyl)-hexoside with molecular formula C_24_H_28_O_14_, which yielded a deprotonated ion at *m*/*z* 539.1377 and generated the following fragment ions: *m*/*z* at 359.09 ([M-H-syringoyl moiety]), 341.08 ([M-H-syringoyl moiety-H_2_O]), 197.04 (syringic acid), and 153.05 because of the loss of water molecule from ion *m*/*z* 197.04 (Figure 3B). Compound **9** too has been identified for the first time in RADP.

#### 2.6.2. Flavonoids

A previous review demonstrated that each subgroup of flavonoids exhibits a different fragmentation behavior in MS^2^ analysis. The cleavage of the C-ring bonds (retro-Diels-Alder, i.e., RDA mechanism) produces ions with the A- or B-ring and some part of the C-ring, which is the most common fragmentation of flavonoids [47]. Notable losses of small neutral molecules, such as CO (28 Da), C_2_H_2_O (42 Da), COO (44 Da), 2CO (56 Da), CO + COO (72 Da), and 3CO (84 Da), may also occur [48]. Based on a comparison of the fragmentation patterns with those previously published in the literature, compounds **10**, **11**, and **20** were identified as chrysoeriol, quercetin, and afzelin gallate, respectively [1,17,40]. Along with their antioxidant properties, quercetin, and chrysoeriol have shown potent anti-cancer, anti-inflammatory, antidiabetic, and antihyperlipidemic effects [49,50]. Flavonoids are frequently glycosylated. The typical fragmentation of *O*-glycosides produces neutral species corresponding to sugar units (hexoses, 162 Da; deoxyhexoses, 146 Da; pentoses, 132 Da) and an aglycone ion. Conversely, C-glucosides produce a sequence of fragments because of the cleavage of the C-C bonds with the sugar moiety; examples of such fragments are [M-H-60]^−^, [M-H-90]^−^, and [M-H-120]^−^, which serve as the hallmark diagnostic ions of glycone [51,52]. Compounds **12**–**18** and **21**–**29** were identified as dihydrokaempferol hexoside (aromadendrin hexoside), isoquercitrin, isorhamnetin hexoside, luteolin hexosyl sulfate, chrysoeriol hexosyl sulfate, isoquercitrin sulfate, apigenin-8-C-(pentosyl)-hexoside (vitexin rhamnoside), luteolin rhamnosyl hexoside, chrysoeriol rhamnosyl hexoside (chrysoeriol rutinoside), quercetin rhamnosyl glucoside (rutin), isorhamnetin rhamnosyl glucoside (isorhamnetin rutinoside), quercetin diglucoside, isorhamnetin diglucoside, luteolin dihexosyl sulfate, luteolin rhamnosyl dihexoside (luteolin rutinoside), and chrysoeriol rhamnosyl dihexoside (chrysoeriol rutinoside), respectively, based on the similarities observed in the comparison of their fragmentation behaviors and with the behaviors reported in the literature [1,13,17,40]. The flavonoid glycosides’ DPPH-scavenging action is as follows: isorhamnetin rutinoside > isoquercitrin > isorhamnetin hexoside > chrysoeriol rutinoside > rutin > vitexin rhamnoside ≅ luteolin rutinoside [53,54,55,56]. The deprotonated molecular ion [M–H]^−^ at *m*/*z* 581.1863 exhibited MS^2^ fragment ions at *m*/*z* 436.13 and 274.08 by loss of rhamnosyl (145 Da) and rhamnosyl-glucosyl moieties (309 Da). The ion at *m*/*z* 274.08 further yielded MS^3^ ion at *m*/*z* 167.03 and 149.06 through cleavage of the α-β bond and loss of the phloroglucinol moiety (125 Da); hence, compound **19** was tentatively identified as naringin dihydrochalcone, which has been identified for the first time in RADP (Figure 3C). Naringin dihydrochalcone has more excellent antioxidant activity than naringin [57]. It was proposed as a potential therapeutic agent for Alzheimer’s disease treatment due to its ability to combat several effects that increase neurogenesis, suppress neuroinflammation, and decrease Aβ levels [58].

#### 2.6.3. Lignans

**Figure 4:**
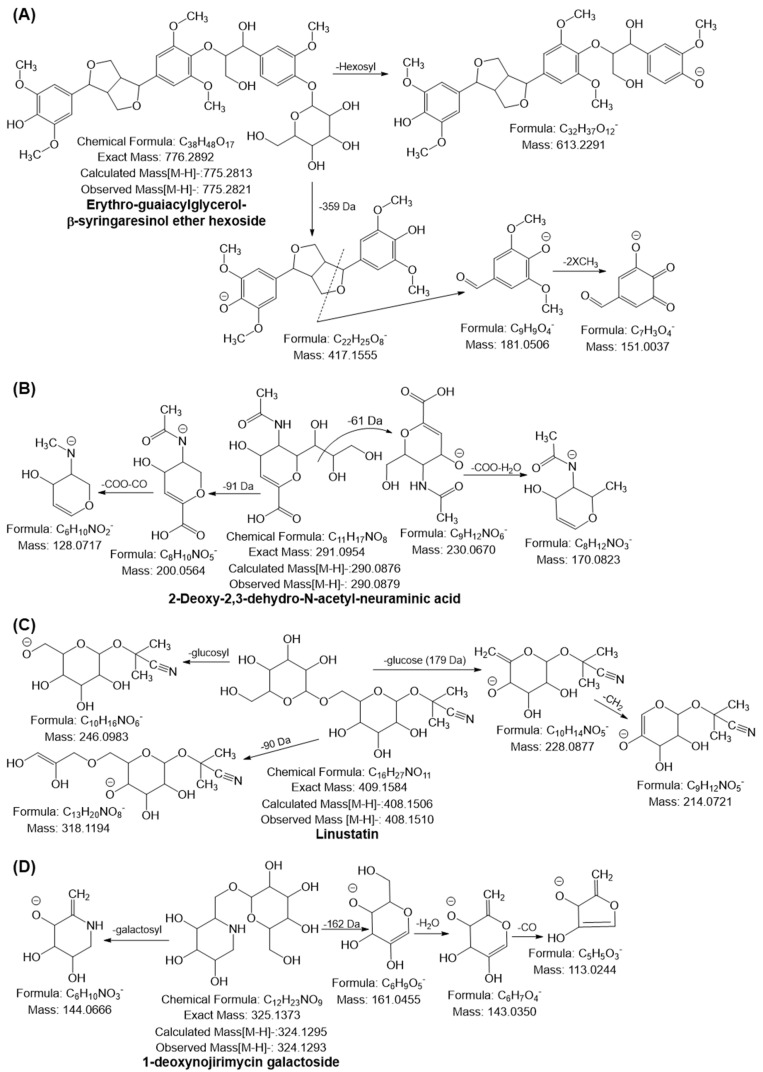
Possible mass fragmentation behavior of identified compounds in RADP. (**A**) erythron-guaiacylglycerol-β-syringaresinol ether hexoside, (**B**) 2-deoxy-2,3-dehydro-N-acetyl-neuraminic acid, (**C**) linustatin, and (**D**) 1-deoxynojirimycin galactoside.

The monoisotopic mass [M–H]^−^ at *m*/*z* 775.2821 yielded a characteristic fragment ion at *m*/*z* 613 by loss of hexosyl moiety (162 Da) and *m*/*z* 417.155, confirming the presence of syringaresinol. The MS^2^ ion at *m*/*z* 417.15 underwent further cleavage of the furfuran ring followed by the loss of two molecules of CH_3_ generated ion at *m*/*z* 181.05 and 151.00, respectively; compound **30** was tentatively confirmed as a furfuran-type lignan compound, namely erythro-guaiacylglycerol-β-syringaresinol ether hexoside (Figure 4A). Furthermore, the deprotonated ion [M–H]^−^ of compound **31** was obtained at *m*/*z* 805.2926, and it yielded a fragment ion at *m*/*z* 643.23 by the loss of the hexosyl moiety, which was 31 Da higher than that of compound **30**. This suggests that the compound has one more methoxy group. The other fragmentation behavior observed was similar to that of the quasimolecular ion of compound **30**, suggesting that compound **31** was erythro-1-(4′-*O*-hexoside-3,5-dimethoxyphenyl)-syringaresinoxyl-propane-1,3-diol (supplementary data Figure S5B). Both lignans were identified for the first time in RADP. The biological activities of these lignans remain unknown [59,60].

#### 2.6.4. Sialic Acid and Derivatives

The deprotonated ion [M–H]^−^ of No. **32** was observed at *m*/*z* 290.0879, and diagnostic ions were generated at *m*/*z* 230.06 and 200.05 by the loss of 61 and 91 Da, respectively. Both MS^2^ ions further generated fragment ions at *m*/*z* 146.08 ([M-H-230.06-COO-H_2_O]) and 128.07 ([M-H-200.05-COO-CO]), suggesting that the compound was 2-deoxy-2,3-dehydro-N-acetyl-neuraminic acid (DANA) (Figure 4B). The monoisotopic mass [M–H]^−^ at *m*/*z* 308.0987 and 632.2039 with molecular formulas C_11_H_19_NO_9_ and C_23_H_39_NO_19_ suggested that compounds **33** and **34** were N-acetyl-neuraminic acid and 3′-sialyllactose (SL), respectively, as their mass fragmentation behaviors were quite similar to the behavior of the quasimolecular ion of compound **32** (supplementary data Figure S5C,D). This is the first time that sialic acid has been identified in RADP. DANA is a glucose-dependent potentiator of insulin secretion [61]. The positive effects of SL on inflammation and immunological homeostasis by altering the gut microbiota profile have been documented in numerous investigations. It also slows the progression of rheumatoid arthritis by reducing chemokines and cytokines, and it relieves osteoarthritis by promoting cartilage regeneration and preventing cartilage from being destroyed [62,63,64].

#### 2.6.5. Amino Acids, Carboxylic Acids, and Fatty Acids

From comparisons of the mass and the fragmentation behaviors of the precursor ion based on mass spectroscopic analysis reported in the literature and various online databases, compounds **35**–**38**, **48**–**55**, and **56**–**67** were identified as amino acids, carboxylic acids, and fatty acids, respectively (Table 5) [17,40,47,48,65,66,67]. Pyroglutamic acid is utilized as a humectant in products for dry skin and hair and has potential anti-angiotensin-converting enzyme activity [68].

#### 2.6.6. Sugar Molecules

From the monoisotopic ion [M–H]^―^ at *m*/*z* 351.0569, the diagnostic ions yielded at *m*/*z* 291.07 ([M-H-COO-H_2_O]), 273.06 ([M-H-291.07-H_2_O]), 175.02 ([M-H-glucoronyl]), and 131.03 ([M-H-175.02-COO]). Compound **46** was tentatively identified as unsaturated digalacturonate; this is the first time that this compound was found in RADP (supplementary data Figure S6A). Furthermore, compound **47** was tentatively identified as xylosmaloside with the molecular formula C_18_H_20_O_9_, and this compound generated the deprotonated ion [M–H]^−^ at *m*/*z* 379.1027 and the following mass fragmentation pattern: *m*/*z* 343.08 ([M-H-36 Da]), 217.05 ([M-H-162 Da]), 179.05 (xylose), and 161.04 ([M-H-179.05-18 Da]) (supplementary data Figure S6B). This compound too was identified for the first time in RADP. Compounds **39**–**45** were confirmed as sugar molecules from comparison of their deprotonated ion mass and fragmentation behaviors with those reported in the literature and online databases [47,48,65,66,67,69]. Maltitol, one of the sugar molecules, inhibits *Streptococcus mutans* DMST 18777, and xylosmaloside has greater antioxidant properties than ascorbic acid [70,71].

#### 2.6.7. Others

Compound **68** yielded a monoisotopic mass [M–H]^−^ at *m*/*z* 408.1510 and generated a diagnostic ion at *m*/*z* 246.09 by loss of the glucosyl moiety (162 Da). Furthermore, a prominent ion peak was observed at *m*/*z* 228.08 with the neutral loss of glucose moiety. In the MS^3^ spectra of *m*/*z* 228, ions at *m*/*z* 214.07 were observed owing to the loss of CH_2_, suggesting that the compound was linustatin, a cyanogenic glucoside, which has also been identified for the first time in RADP (Figure 4C). The deprotonated ion [M–H]^−^ at *m*/*z* 324.1293 and the characteristic ions at *m*/*z* 144.06 ([M-H-galactosyl moiety]) and 161.04 ([M-H-162 Da]) were observed. The fragment ion at *m*/*z* 161.04 further yielded ions at *m*/*z* 143.03 and 113.02 through the consecutive loss of H_2_O and CO. Based on the mass fragmentation behavior, compound **69** was tentatively identified, for the first time in RADP, as 1-deoxynojirimycin galactoside (Figure 4D). Although 1-deoxynojirimycin galactoside’s pharmacological role is unknown, its aglycone component (1-deoxynojirimycin) has a powerful antihyperglycemic and anti-obesity activity [72]. Compound **70** was tentatively identified as oxycoumarin-4-acetic acid methyl ether hexoside, with the molecular formula C_18_H_20_O_10_, and it yielded a deprotonated ion [M–H]^−^ at *m*/*z* 395.0968. It gave a prominent peak at *m*/*z* 233.04, owing to the loss of galacotosyl moiety (162 Da). In the MS^3^ spectra of 233.04, ions at *m*/*z* 205.05 and 161.02 were generated through the loss of CO and acetic acid methyl ether moiety (supplementary data Figure S6C).

## 3. Materials and Methods

### 3.1. Sample Collection and Preparation

Ripe Ajwa dates were collected from an Ajwa date farm located in Medina, Saudi Arabia, and were identified by a scientific officer at the National Herbarium and Genebank of Saudi Arabia. A voucher specimen (No. NHG005) has been stored in our laboratory for further investigation. Heat extraction (HE) was performed in a temperature-controlled heater using a round-bottom flask with a condenser to prevent solvent evaporation (Soxlet water bath C-WBS-D6, Changshin Science, Seoul, Republic of Korea). The dry powder samples (10.0 g) were extracted thrice with 100 mL solvent according to the investigational design shown in supplemental data Tables S1 and S2. After extraction, the samples were filtered using Whatman No. 1 filter paper (Schleicher & Schuell, Keene, NH, USA), concentrated to dryness in a rotary evaporator (Tokyo Rikakikai Co. Ltd., Tokyo, Japan) at 40 °C and 50 rpm, and then lyophilized using a freeze-dryer (Il-shin Biobase, Goyang, Republic of Korea). The RADP extract was kept at −20 °C before subsequent experiments.

### 3.2. Antioxidant Activities

The total phenolic content (TPC) and total flavonoid content (TFC) in ripe Ajwa date pulp (RADP) extracts were determined using the Folin–Ciocalteu test and the aluminum chloride colorimetric method, respectively [45]. The TPC (y = 0.0512x + 0.0018; r^2^ = 0.9835) and TFC (y = 0.014x + 0.0021; r^2^ = 0.9994) were determined using the corresponding regression equations for the calibration curves. The TPC was expressed in terms of the gallic acid equivalent (mg)/dry weight sample (g), and the TFC in terms of the catechin equivalent (mg)/dry weight sample (g). The DPPH radical scavenging assay and the cupric-reducing antioxidant capacity (CUPRAC) assay were applied to assess the antioxidant activity of RADP extracts [28].

### 3.3. Experimental Design of Response Surface Methodology (RSM) for the Extraction Process

The RSM model was developed for the extraction of phenolic compounds from RADP; the independent process factors were ethanol concentration (X_1_), time (X_2_), and temperature (X_3_), and the response variables were TPC (Y_1_), TFC (Y_2_), DPPH-scavenging activity (Y_3_), and CUPRAC (Y_4_). The extractions were carried out using a central composite design (CCD) with three components and five layers. The response variables were fitted to the second-order polynomial model equation shown in Equation (5), which specifies the relationship between the independent variables and responses.

(5) $$Y=\beta_{0}+\sum_{i=1}^{n}\beta_{i}X_{i}+\sum_{i=1}^{n}\beta_{ii}X_{ii}^{2}+\sum_{i}^{n-1}\sum_{j}^{n}\beta_{ij}X_{ij}$$

where **Y** is the response variable, and *X_i_* and *X_j_* are the independent coded variables; *β_0_* denotes the constant coefficient, and *β_i_, β_ii_,* and *β_ij_* denote the coefficients of linear, quadratic, and interaction effects, respectively. Design Expert 11 was used for the RSM analysis and multiple linear regression (Stat-Ease, Minneapolis, MN, USA). To select the best model, the function parameter was selected as “None” and various R^2^ (coefficient of determination) values, PRESS (the predicted residual sum of squares), BIC (the Bayesian information criterion), and AICc (the Akaike information criterion–second-order) were considered. The F-values with *p* < 0.05 indicated statistical significance. The interaction outcome of each factor on the response value was represented in the form of three-dimensional (3D) surface plots.

### 3.4. Artificial Neural Networks (ANN) Modeling

**Figure 5:**
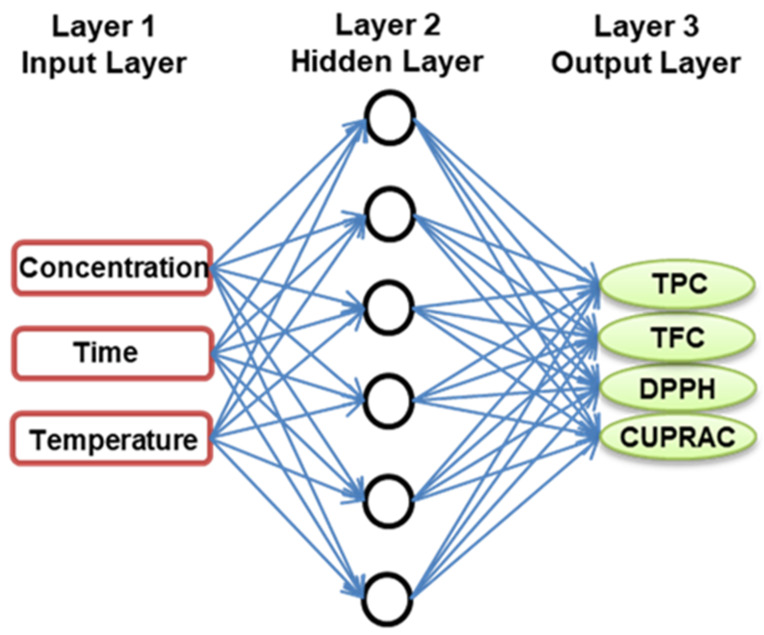
Optimal topology of a developed ANN model.

An ANN was used to predict a nonlinear relationship between RADP’s input parameters (X_1_, X_2_, and X_3_) and response variables (Y_1_, Y_2_, Y_3_, and Y_4_). The Neural Network ToolboxTM of MATLAB was used for the nonlinear analysis, and the multilayer perceptron (MLP) was utilized to map layers of independent and response variables using a back-propagation feed-forward ANN model [28]. The hit-and-trial method was used to calculate the required number of neurons in the hidden layer, which ranged from 1 to 15, to minimize discrepancies between the predicted and experimental results of RADP. Figure 5 depicts the three-layered fundamental architecture (MLP topology) of the ANN model. The model’s fundamental architecture consists of three layers: a three-neuron input layer representing the three independent factors (ethanol concentration, extraction time, and temperature); a six-neuron hidden layer; and an output layer representing the four response variables (TPC, TFC, DPPH-scavenging activity, and CUPRAC value) of RADP. The investigational dataset used to create the RSM model was also used to develop an ANN model: network training (70%: 20 points), validation (15%: 5 points), and network testing (15%: 5 points). In the ANN design, the output signal was made by sending the weighted sum of the input variables to each neuron through an activation function. This function was usually nonlinear and was represented by the hidden layer.

### 3.5. Comparison of the Prediction Ability of the RSM and ANN Models

The following equations (Equations (6)–(9)) were used to calculate various statistical parameters, including the correlation coefficient (*R*^2^), root-mean-square error (*RMSE*), absolute average deviation (*AAD*), and standard error of prediction (*SEP*), to compare the estimation capabilities of RSM and ANN for improving the extraction procedure with better TPC, TFC, and antioxidant potential of RADP.

(6) $$R^{2}=1-\frac{\sum_{i=1}^{n}{\left(Y_{p}-Y_{e}\right)}^{2}}{\sum_{i=1}^{n}{\left(Y_{m}-Y_{e}\right)}^{2}}$$

(7) $$RMSE=\sqrt{\frac{\sum_{i=1}^{n}{\left(Y_{p}-Y_{e}\right)}^{2}}{n}}$$

(8) $$AAD=\left[\frac{\sum_{i=1}^{n}\left(\left|Y_{p}-Y_{e}\right|/Y_{e}\right)}{n}\right]\;\times \;100$$

(9) $$SEP=\frac{RMSE}{Y_{m}}\;\times \;100$$

where *Y_p_* is the predicted response; *Y_e_* is the observed response; *Y_m_* is the average response variable; n is the number of experiments.

### 3.6. Validation of the Model

Derringer’s desire function was used to find the ideal conditions for maximizing all replies in a single experiment. Each response is turned into a unique desirability function ranging from 0 to 1 during this procedure (in the order of the lowest to the highest desirability). The component functions are then combined to create a total desirability function. The total desirability function is constructed using the following equation [1].

(10) $$D={\left(d_{1}^{w1}d_{2}^{w2}\ldots .d_{n}^{wn}\right)}^{1/\sum^{\;}wi}$$

Response surface and desirability function analyses were used to determine the optimal RADP extraction parameters, which were 75% ethanol concentration, 3.7 h of extraction time, and 67 °C temperature with D = 0.934. A triple experiment was conducted under the best possible circumstances to validate the existing model. The average experimental results were then compared to the predicted results. The experimental data were also compared to the values that the model indicated. To compare the observed and anticipated outcomes of RADP, Equation (11) was utilized to calculate the relative standard deviation (*RSD*).

(11) $$\;\;\;\;RSD\;\left(\%\right)=\frac{Standard\;deviation\;between\;predicted\;and\;experimental\;values}{Mean\;values\;between\;predicted\;and\;experimental\;values}\;\times \;100$$

The resulting data were analyzed and optimized for all response circumstances when the *RSD*% was <10. Additionally, the electrospray ionization mass spectrometry (ESI-MS)/MS profiles of bioactive compounds were found under ideal circumstances of RADP.

### 3.7. Analysis of Chemical Compounds by ESI-MS/MS

The Q-Exactive Orbitrap mass spectrometer (Thermo Fisher Scientific INC., San Jose, CA, USA) was used to conduct the positive (+) and negative (−) mode ESI-MS investigations of RADP. A 500 μL graded syringe (Hamilton Company Inc., Reno, NV, USA) and a 15 μL/min syringe pump (Model 11, Harvard, Holliston, MA, USA) were used to immerse the RADP in the ESI source. The normal negative mode ESI-MS conditions were as follows: mass resolution of 140,000 (full width at half maximum, FWHM), sheath gas flow rate of 5, seep gas flow rate of 0, auxiliary gas flow rate of 0, spray voltage of 4.20 kV, capillary temperature of 320 °C, S-lens Rf level, and automatic gain control of 5 E 6. The MS/MS experiments were conducted on the same instrument utilizing three unique stepwise normalized collision energies (10, 30, and 40) to produce better fragmentations of each peak, hence facilitating the confirmation of RADP for each compound [48].

The Xcalibur 3.1 with Foundation 3.1 (Thermo Fisher Scientific Inc. Rockford, IL, USA) was used to process the collected mass spectral data. The *m*/*z* peaks were tentatively identified by comparing their calculated (exact) masses of protonated and/or deprotonated (M–H) adducts with the *m*/*z* values and ESI-MS/MS fragmentation patterns from the in-house MS/MS database and online databases such as METLIN [65], CFM-ID 4.0 [66] and FooDB [67]. The chemical structure was drawn using ChemDraw Professional 15.0 (PerkinElmer, Waltham, MA, USA).

### 3.8. Statistical Analysis

The experimental results were statistically analyzed using Design Expert 11 and MATLAB software. All data were reported as the mean ± standard deviation of at least three independent experiments (n = 3), each with three sample replicates. Differences were considered significant at *p* < 0.001, *p* < 0.01, and *p* < 0.05.

## 4. Conclusions

This work was the first to use two modeling methodologies (RSM and ANN) to optimize the heat extraction (HE) conditions for achieving the highest amount of TPC, TFC, and antioxidant potential of RADP. The ANN model is more accurate and reliable for optimizing the extraction conditions of RADP for improved attainment of TPC, TFC, and antioxidant characteristics, as evidenced by the greater R^2^ and lower RMSE, AAD, and SEP compared to the RSM model. The optimal conditions (75% ethanol, extraction time of 3.7 h, and extraction temperature of 67 °C) were determined to maximize the TPC, TFC, and antioxidant potential of RADP. Under optimum conditions, TPC, TFC, DPPH radical scavenging effect, and ascorbic acid equivalent CUPRAC value were obtained as 4.53 mg GAE/g, 3.30 mg CAE/g, 10.69% inhibition, and 1.41 μM, respectively. RADP contains phenolic acids, flavonoids, sialic acids, lignans, etc., as determined by high-resolution mass spectrometry analysis. Sialic acids and lignans were described for the first time in RADP as bioactive substances. Based on these data, we can infer that RADP, a viable candidate for an antioxidant functional food, could be used extensively in the nutraceutical and pharmaceutical industries.

## Figures and Tables

**Table 1:** Central composite design (CCD) for independent variables and corresponding response values (experimental).

Run	Independent Variables			Responses			
	(X_1_)	(X_2_)	(X_3_)	TPC (Y_1_)	TFC (Y_2_)	DPPH (Y_3_)	CUPRAC (Y_4_)
1	75	4	70	4.30 ± 0.68	3.16 ± 0.73	10.81 ± 0.72	1.38 ± 0.05
2	25	2	50	3.81 ± 0.83	2.55 ± 0.52	10.25 ± 0.39	1.47 ± 0.02
3	75	2	50	3.90 ± 0.34	2.55 ± 0.25	9.75 ± 0.37	1.16 ± 0.16
4	100	3	60	4.30 ± 0.26	3.44 ± 0.65	10.11 ± 0.24	1.12 ± 0.10
5	75	2	70	3.50 ± 0.41	2.56 ± 0.32	9.95 ± 1.00	1.17 ± 0.07
6	50	3	60	4.53 ± 0.82	3.10 ± 0.21	10.45 ± 0.10	1.40 ± 0.08
7	0	3	60	3.01 ± 1.02	2.52 ± 0.25	9.57 ± 0.56	0.91 ± 0.06
8	75	4	50	4.31 ± 0.49	2.95 ± 0.45	9.88 ± 0.26	0.88 ± 0.04
9	50	3	60	4.30 ± 0.42	3.15 ± 0.54	10.40 ± 0.95	1.46 ± 0.08
10	50	3	60	4.51 ± 0.23	3.09 ± 0.98	10.42 ± 0.35	1.43 ± 0.04
11	50	3	80	3.78 ± 0.62	2.26 ± 0.10	9.50 ± 0.26	1.11 ± 0.02
12	50	3	60	4.46 ± 0.06	3.01 ± 1.02	10.48 ± 0.33	1.45 ± 0.06
13	50	3	60	4.48 ± 0.04	3.12 ± 0.56	10.41 ± 0.53	1.43 ± 0.16
14	25	4	70	3.15 ± 0.08	2.16 ± 0.46	9.60 ± 0.35	1.00 ± 0.13
15	50	3	60	4.51 ± 0.06	2.95 ± 0.29	10.20 ± 0.15	1.45 ± 0.19
16	50	3	40	3.85 ± 0.24	2.48 ± 0.37	9.60 ± 0.60	0.92 ± 0.15
17	25	2	70	3.25 ± 0.68	2.15 ± 0.19	9.36 ± 0.72	1.12 ± 0.07
18	50	1	60	2.87 ± 0.68	2.03 ± 0.49	9.80 ± 0.39	1.32 ± 0.10
19	50	5	60	2.90 ±0.64	2.50 ± 1.05	9.90 ± 0.72	0.86 ± 0.02
20	25	4	50	2.96 ± 0.80	2.38 ± 1.06	9.53 ± 0.39	0.75 ± 0.08

X_1_: ethanol concentration (%); X_2_: time (h); X_3_: temperature (°C). TPC: total phenolic content (mg gallic acid equivalent/g dry weight extract); TFC: total flavonoid content (mg catechin equivalent/g dry weight extract); DPPH: DPPH radical scavenging activity (% inhibition); CUPRAC: cupric-reducing antioxidant capacity (µM ascorbic acid equivalent).

**Table 2:** ANOVA for quadratic model (transfer function: none).

**ANOVA for Quadratic Model for TPC**							
**Source**	**RC**	**SS**	**DF**	**MS**	**F-Value**	***p*-Value**	
Model		7.18	9	0.7975	81.83	<0.0001	Significant
Intercept	4.45						
Linear terms							
X_1_	0.3400	1.85	1	1.85	189.79	<0.0001	Significant
X_2_	0.0200	0.0064	1	0.0064	0.6567	0.4366	Non-significant
X_3_	−0.0575	0.0529	1	0.0529	5.43	0.0421	Significant
Interaction terms							
X_1_X_2_	0.2700	0.5832	1	0.5832	59.84	<0.0001	Significant
X_1_X_3_	−0.0050	0.0002	1	0.0002	0.0205	0.8889	Non-significant
X_2_X_3_	0.1425	0.1625	1	0.1625	16.67	0.0022	Significant
Quadratic terms							
X_1_^2^	−0.2089	1.10	1	1.10	112.55	<0.0001	Significant
X_2_^2^	−0.4001	4.03	1	4.03	413.02	<0.0001	Significant
X_3_^2^	−0.1676	0.7064	1	0.7064	72.48	<0.0001	Significant
Lack of Fit		0.0617	5	0.0123	1.73	0.2819	Non-significant
Pure error		0.0358	5	0.0072			
R^2^							0.9866
Adjusted R^2^							0.9745
Predicted R^2^							0.9262
Adeq Precision							23.5003
C.V. %							2.58
PRESS							0.5369
BIC							−19.77
AICc							−5.28
**ANOVA for quadratic model for TFC**							
Model		3.11	9	0.3454	39.85	<0.0001	significant
Intercept	3.05						
Linear terms							
X_1_	0.2388	0.9120	1	0.9120	105.24	<0.0001	Significant
X_2_	0.1113	0.1980	1	0.1980	22.85	0.0007	Significant
X_3_	−0.0525	0.0441	1	0.0441	5.09	0.0477	Significant
Interaction terms							
X_1_X_2_	0.1450	0.1682	1	0.1682	19.41	0.0013	Significant
X_1_X_3_	0.1050	0.0882	1	0.0882	10.18	0.0097	Significant
X_2_X_3_	0.0475	0.0180	1	0.0180	2.08	0.1795	Non-significant
Quadratic terms							
X_1_^2^	−0.0328	0.0271	1	0.0271	3.13	0.1073	Non-significant
X_2_^2^	−0.2116	1.13	1	1.13	129.89	<0.0001	Significant
X_3_^2^	−0.1853	0.8637	1	0.8637	99.66	<0.0001	Significant
Lack of Fit		0.0585	5	0.0117	2.07	0.2213	Non-significant
Pure error		0.0282	5	0.0056			
R^2^							0.9729
Adjusted R^2^							0.9485
Predicted R^2^							0.8432
Adeq Precision							21.4961
C.V. %							3.44
PRESS							0.5009
BIC							−22.11
AICc							−7.63
**ANOVA for quadratic model for DPPH**							
Model		3.12	9	0.3470	34.85	<0.0001	Significant
Intercept	10.39						
Linear terms							
X_1_	0.1706	0.4658	1	0.4658	46.77	<0.0001	Significant
X_2_	0.0444	0.0315	1	0.0315	3.16	0.1057	Non-significant
X_3_	0.0069	0.0008	1	0.0008	0.0759	0.7885	Non-significant
Interaction terms							
X_1_X_2_	0.1837	0.2701	1	0.2701	27.12	0.0004	Significant
X_1_X_3_	0.2437	0.4753	1	0.4753	47.73	<0.0001	Significant
X_2_X_3_	0.2112	0.3570	1	0.3570	35.85	0.0001	Significant
Quadratic terms							
X_1_^2^	−0.1399	0.4920	1	0.4920	49.40	<0.0001	Significant
X_2_^2^	−0.1374	0.4746	1	0.4746	47.65	<0.0001	Significant
X_3_^2^	−0.2124	1.13	1	1.13	113.88	<0.0001	Significant
Lack of Fit		0.0505	5	0.0101	1.03	0.4887	Non-significant
Pure error		0.0491	5	0.0098			
R^2^							0.9691
Adjusted R^2^							0.9413
Predicted R^2^							0.8504
Adeq Precision							18.9896
C.V. %							0.9981
PRESS							0.4822
BIC							−19.33
AICc							−4.85
**ANOVA for quadratic model for CUPRAC**							
Model		1.10	9	0.1218	154.89	<0.0001	Significant
Intercept	1.43						
Linear terms							
X_1_	0.0419	0.0281	1	0.0281	35.68	0.0001	Significant
X_2_	−0.1144	0.2093	1	0.2093	266.19	<0.0001	Significant
X_3_	0.0481	0.0371	1	0.0371	47.13	<0.0001	Significant
Interaction terms							
X_1_X_2_	0.0963	0.0741	1	0.0741	94.25	<0.0001	Significant
X_1_X_3_	0.0762	0.0465	1	0.0465	59.15	<0.0001	Significant
X_2_X_3_	0.1363	0.1485	1	0.1485	188.87	<0.0001	Significant
Quadratic terms							
X_1_^2^	−0.1074	0.2899	1	0.2899	368.74	<0.0001	Significant
X_2_^2^	−0.0886	0.1975	1	0.1975	251.22	<0.0001	Significant
X_3_^2^	−0.1086	0.2967	1	0.2967	377.37	<0.0001	Significant
Lack of Fit		0.0055	5	0.0011	2.37	0.1827	Non-significant
Pure error		0.0023	5	0.0005			
R^2^							0.9929
Adjusted R^2^							0.9865
Predicted R^2^							0.9580
Adeq Precision							34.9882
C.V. %							2.36
PRESS							0.0464
BIC							−70.11
AICc							−55.62

RC: regression coefficient; SS: sum of squares; MS: mean square.

**Table 3:** Comparison of the prediction abilities of the RSM and ANN models.

Parameters	TPC		TFC		DPPH		CUPRAC	
	RSM	ANN	RSM	ANN	RSM	ANN	RSM	ANN
R^2^	0.9866	0.9899	0.9729	0.9859	0.9691	0.9828	0.9929	0.9954
RMSE	0.8053	0.4475	0.1663	0.1134	0.2586	0.1635	0.1063	0.0903
AAD (%)	4.079	2.052	6.011	3.753	0.8815	0.7011	6.764	3.738
SEP (%)	0.2225	0.1518	0.4591	0.2903	0.0529	0.0449	0.3710	0.2728

**Table 4:** Experiment data of the validation of predicted values at optimal extraction conditions of RADP.

Response	Exp.	Pred.	Std	RSD (%)
TPC (mgGAE/g)	4.49 ± 1.02	4.53	0.028	0.006
TFC (mgCAE/g)	3.31 ± 0.65	3.30	0.007	0.002
DPPH (% inhibition)	11.10 ± 0.78	10.69	0.290	0.027
CUPRAC (μM ASCE)	1.43 ± 0.43	1.41	0.014	0.010

Optimal condition: ethanol concentration (%), 75 %; time (h), 3.7; temperature (°C), 67. Exp.: experimental value; Pred.: predicted value; Std: standard deviation; RSD: relative standard deviation.

**Table 5:** List of possible identified compounds of the optimized extract of ripe Ajwa date pulp (RADP) by electrospray ionization mass spectrometry (ESI-MS)/MS.

	No.	Compound Name	EF	OM (*m*/*z*)^−/+^	CM (*m*/*z*)^−/+^	MS/MS (Negative Mode)	CE	CL
Phenolic acids and derivatives	1	Coumalic acid ^#^	C_6_H_4_O_4_	139.0050	139.0031	111.01, 95.01	20	3
	2	Hydroxybenzoylhexose	C_13_H_16_O_8_	299.0876	299.0884	281.06, 237.04, 179.03, 163.06, 137.02	20	2
	3	Coumaroyl hexose	C_15_H_18_O_8_	325.0929	325.0923	163.03, 147.04	10	2
	4	Caffeoylshikimic acid	C_16_H_16_O_8_	335.0776/337.0932	335.0772	179.01, 161.03, 155.03, 137.05	20	2
	5	Caffeic acid hexoside	C_15_H_18_O_9_	341.1100	341.0872	215.03, 179.06, 161.04	20	2
	6	Caffeic acid derivatives	C_18_H_18_O_9_	377.0885	377.0878	341.10, 215.03, 179.06, 161.04	10	2
	7	Dicaffeoyl shikimic acid	C_22_H_26_O_13_	497.1297	497.1295	335.01, 178.02, 135.02	20	2
	8	Hebitol II ^#^	C_21_H_30_O_14_	505.1603	505.1557	341.08,325.09, 179.03, 163.03	30	3
	9	1,2-di-(syringoyl)-hexoside ^#^	C_24_H_28_O_14_	539.1377/541.1533	539.1401	359.09, 341.08, 197.04, 153.05	30	3
Flavonoids and derivatives	10	Chrysoeriol	C_13_H_16_O_8_	299.0561/301.0717	299.0555	285.03, 255.02, 153.01, 147.04, 135.03, 125.03	20	2
	11	Quercetin	C_15_H_10_O_7_	301.0354/303.0510	301.0348	273.02, 257.03, 229.05, 179.01, 151.01	20	2
	12	Dihydrokaempferol hexoside	C_21_H_22_O_11_	449.1089	449.1083	287.04, 269.05, 259.06, 169.01, 151.01	20	2
	13	Isoquercitrin	C_21_H_20_O_12_	463.0878	463.0876	301.05, 268.01, 179.02, 151.01	20	2
	14	Isorhamnetin hexoside	C_22_H_22_O_12_	477.1035/479.1191	477.1033	315.05, 300.01, 271.02, 255.05, 179.05, 151.02	20	2
	15	Luteolin hexosyl sulfate	C_21_H_20_O_14_S	527.0491/529.0647	527.0495	447.05, 285.01, 241.06	20	2
	16	Chrysoeriol hexosyl sulfate	C_22_H_22_O_14_S	541.0645/543.0801	541.0652	299.05, 284.05, 241.02	20	2
	17	Isoquercitrin sulfate	C_21_H_20_O_15_S	543.0441/545.0597	543.0444	463.05, 301.01, 268.01, 179.02, 151.01	20	2
	18	Apigenin-8-C-(pentosyl) hexoside	C_26_H_28_O_14_	563.1655	563.1400	473.01, 443.02, 413.05, 340.08, 311.02	30	2
	19	Naringin dihydrochalcone ^#^	C_27_H_34_O_14_	581.1863	581.1870	436.13, 274.08, 167.03, 149.06, 133.06,	30	3
	20	Afzelin gallate	C_28_H_24_O_14_	583.1093	583.1087	297.05, 285.04, 169.01	20	2
	21	Luteolin rhamnosyl hexoside	C_27_H_30_O_15_	593.1507	593.1506	447.09, 285.03, 153.01, 135.04	20	2
	22	Chrysoeriol rhamnosyl hexoside	C_28_H_32_O_15_	607.1669	607.1663	461.10, 299.05, 284.03, 153.01, 149.05	20	2
	23	Quercetin rhamnosyl glucoside	C_27_H_30_O_16_	609.1459	609.1455	463.08, 447.09, 301.02, 151.04	20	2
	24	Isorhamnetin rhamnosyl glucoside	C_28_H_32_O_16_	623.1617/625.1773	623.1612	477.10, 315.05, 299.05, 165.05	20	2
	25	Quercetin diglucoside	C_27_H_30_O_17_	625.1410/627.1566	625.1404	463.08, 301.01	20	2
	26	Isorhamnetin diglucoside	C_28_H_32_O_17_	639.1563/641.0612	639.1561	447.01, 315.01	20	2
	27	Luteolin dihexosyl sulfate	C_27_H_30_O_19_S	689.1029	689.1023	519.11, 489.10, 471.09, 399.07, 369.06, 339.05	20	2
	28	Luteolin rhamnosyl dihexoside	C_33_H_40_O_20_	755.2046	755.2034	709.16, 593.10, 575.05, 285.01	20	2
	29	Chrysoeriol rhamnosyl dihexoside	C_34_H_42_O_20_	769.2189	769.2191	623.16, 461.10, 299.05, 284.03, 153.02	20	2
Lignans	30	Erythro-guaiacylglycerol-β-syringaresinol ether hexoside ^#^	C_38_H_48_O_17_	775.2821	775.2813	613.22, 417.14, 181.05, 151.03	30	3
	31	Erythro-1-(4”-glucoside-3,5-dimethyoxyphenyl)-2-syringaresinoxyl-propane-1,3-diol ^#^	C_39_H_50_O_18_	805.2926	805.2919	643.23, 417.14, 181.05, 151.03	30	3
Sialic acids	32	2-Deoxy-2,3-dehydro-N-acetylneuraminic acid ^#^	C_11_H_17_NO_8_	290.0879	290.0876	230.06, 200.05, 171.01, 128.07	20	3
	33	N-acetyl-α-neuraminic acid ^#^	C_11_H_19_NO_9_	308.0987/310.1143	308.0987	290.09, 219.06, 200.05, 146.08, 128.07	20	3
	34	6′-Sialyllactose ^#^	C_23_H_39_NO_19_	632.2039	632.2044	290.09, 200.05, 128.07	30	3
Amino acids	35	L-proline	C_5_H_9_NO_2_	114.0570	114.0561	70.06	10	2
	36	Pyroglutamic acid	C_5_H_7_NO_3_	128.0360/130.0416	128.0353	82.3, 71.9	10	2
	37	L-aspartic acid	C_4_H_7_NO_4_	132.0329	132.0302	116.03, 88.04	10	2
	38	Allysine ^#^	C_6_H_11_NO_3_	144.0682	144.0666	127.04, 126.05, 100.07	20	3
Sugar molecules	39	Ribonic acid	C_5_H_10_O_6_	165.0421	165.0418	149.04, 105.01, 87.00, 75.00	10	2
	40	L-Galactose	C_6_H_12_O_6_	179.0572	179.0561	161.04, 143.03, 113.02, 101.02,	10	2
	41	Mannitol	C_6_H_14_O_6_	181.0725	181.0718	165.01, 147.03, 129.05, 111.00	20	2
	42	Gluconic acid	C_6_H_12_O_7_	195.0522	195.0504	177.05, 159.02, 129.05, 98.90	10	2
	43	Sedoheptulose	C_7_H_14_O_7_	209.0679	209.0680	191.05, 179.05, 149.04,	20	2
	44	Hexose derivative	C_12_H_19_O_10_	323.0977	323.0978	179.05, 161.04, 143.03, 113.02, 101.02	10	2
	45	Maltitol	C_12_H_24_O_11_	343.1255	343.1240	283.10, 265.09, 179.05, 161.04, 143.03	20	2
	46	Unsaturated digalacturonate ^#^	C_12_H_16_O_12_	351.0574/353.0730	351.0569	291.07, 273.06, 175.02, 131.03	20	3
	47	Xylosmaloside ^#^	C_18_H_20_O_9_	379.1027/381.1183	379.1029	343.08, 217.05, 179.05, 161.04	20	3
Carboxylic acids	48	Fumaric acid	C_4_H_4_O_4_	115.0050	115.0037	71.01	10	2
	49	Glutaconic acid	C_5_H_6_O_4_	129.0203	129.0203	111.00, 85.02	10	2
	50	Glutaric acid	C_5_H_8_O_4_	131.0355	131.0350	113.00, 87.02	10	2
	51	3-Methylglutaconic acid	C_6_H_8_O_4_	143.0367	143.0361	99.03	20	2
	52	Methyl glutaric acid	C_6_H_10_O_4_	145.0521	145.0506	127.02, 101.02	10	2
	53	2-Hydroxyglutaric acid	C_5_H_8_O_5_	147.0301	147.0299	129.01, 99.03	10	2
	54	Hydroxymethyl glutaric acid	C_6_H_10_O_5_	161.0459	161.0455	143.03, 117.05, 99.04	10	2
	55	Citric acid	C_6_H_8_O_7_	191.0197/193.0353	191.0197	173.00, 129.01, 111.00	20	2
Fatty acids	56	Palmitic acid	C_16_H_32_O_2_	255.2330	255.2330	237.23, 211.24, 197.22	20	2
	57	Linolenic acid	C_18_H_30_O_2_	277.2165	277.2169	259.20, 233.22, 205.21, 179.25, 165.23	10	2
	58	α-Linoleic acid	C_18_H_32_O_2_	279.2331	279.2330	261.22	10	2
	59	Oleic acid	C_18_H_34_O_2_	281.2487	281.2486	263.25, 181.21, 127.25	10	2
	60	Hydroxy octadecatrienoic acid ^#^	C_18_H_30_O_3_	293.2120	293.0216	275.22	20	3
	61	Hydroxy octadecadienoic acid	C_18_H_32_O_3_	295.2276	295.2273	277.23	20	2
	62	Hydroxy octadecenoic acid	C_18_H_34_O_3_	297.2433	297.2429	279.23	20	2
	63	Dihydroxy octadecadienoic acid	C_18_H_32_O_4_	311.2246/313.2402	311.2239	293.22, 275.23	20	2
	64	Dihydroxy octadecenoic acid	C_18_H_34_O_4_	313.2381/315.2537	313.2378	295.23, 277.25, 183.32	20	2
	65	Dihydroxy octadecanoic acid	C_18_H_36_O_4_	315.2538/317.2694	315.2535	297.23, 279.25	20	2
	66	Trihydroxy octadecadienoic acid	C_18_H_32_O_5_	327.2176	327.2171	309.23, 291.25, 273.23	20	2
	67	Trihydroxy octadecenoic acid	C_18_H_34_O_5_	329.2346/331.2502	329.2333	311.25, 293.26, 275.23	20	2
Others	68	Linustatin ^#^	C_16_H_27_NO_11_	408.151/410.1666	408.1506	318.11, 246.09, 228.08, 214.07	20	3
	69	Norbellidifodin ^#^	C_13_H_8_O_6_	259.024/261.0396	259.0248	241.01, 215.12, 187.05, 171.03	30	3
	70	1-Deoxynojirimycin hexoside ^#^	C_12_H_23_NO_9_	324.1293	324.1295	161.04, 144.06, 143.03, 113.02	30	3
	71	Oxycoumarin-4-acetic acid methyl ester hexoside ^#^	C_18_H_20_O_10_	395.0962	395.0978	233.04, 205.05, 161.02, 133.02	30	3

EF: elemental formula; OM: observed mass; CM: calculated mass; CE: Collision Energy (eV); CL: confidence level; ^−/+^: negative mode/positive mode. ^#^ First time identification in Ajwa date fruits.

## Data Availability

Not applicable.

## Supplementary Materials

The following supporting information can be downloaded at: https://www.mdpi.com/article/10.3390/ph16020319/s1. Figure S1: The R^2^ value of training, validation, test, and overall for TPC during ANN. Figure S2: The R^2^ value of training, validation, test, and overall for TFC during ANN. Figure S3: The R^2^ value of training, validation, test, and overall for DPPH during ANN. Figure S4: The R^2^ value of training, validation, test, and overall for CUPRAC during ANN. Figure S5: Possible mass fragmentation behavior of identified compounds in RADP. (A) Coumalic acid, (B) Erythro-1-(4”-O-β-D-glucopyranoside-3,5-dimethyoxyphenyl)-2-syringaresinoxyl-propane-1,3-diol, (C) N-acetyl-α-neuraminic acid, and (D) 6′-sialyllactose. Figure S6: Possible mass fragmentation behavior of identified compounds in RADP. (A) Unsaturated digalacturonate, (B) Xylosmaloside, and (C) Oxycoumarin-4-acetic acid methyl ester hexoside. Table S1: Central composite design (CCD) for the independent variables and corresponding response value in RSM and ANN (predicted). Table S2: Independent process variables with experimental ranges and levels for heat reflux extraction of ripe Ajwa date pulp (RADP).
