# Supplementary material for: Profiling of Secondary Metabolites of Optimized Ripe Ajwa Date Pulp (Phoenix dactylifera L.) Using Response Surface Methodology and Artificial Neural Network
Source: Pharmaceuticals (Basel). 2023 Feb 20;16(2):319. doi: 10.3390/ph16020319 (PMC9961821; doi:10.3390/ph16020319)
Supplement: Supplementary file 1 [file pharmaceuticals-16-00319-s001.zip › pharmaceuticals-2175525-supplementary.pdf]

Table S1: Central composite design (CCD) for the independent variables and corresponding response value in RSM and ANN (predicted)

Run	Independent variables			Responses (predicted)							
	(X_1)	(X_2)	(X_3)	(Y_1)		(Y_2)		(Y_3)		(Y_4)	
				RSM	ANN	RSM	ANN	RSM	ANN	RSM	ANN
1	75	4	70	4.30	4.41	3.16	3.19	10.81	10.77	1.38	1.24
2	25	2	50	3.81	3.59	2.55	2.65	10.25	10.24	1.47	1.42
3	75	2	50	3.90	4.11	2.55	2.58	9.75	9.72	1.16	0.98
4	100	3	60	4.30	4.27	3.44	3.11	10.11	10.73	1.12	0.96
5	75	2	70	3.50	3.49	2.56	2.49	9.95	9.95	1.17	1.45
6	50	3	60	4.53	4.34	3.10	2.97	10.45	10.36	1.40	1.37
7	0	3	60	3.00	3.09	2.52	2.96	9.57	9.79	0.91	0.96
8	75	4	50	4.31	3.08	2.95	3.32	9.88	9.9	0.88	0.88
9	50	3	60	4.30	4.34	3.15	2.97	10.40	10.36	1.46	1.37
10	50	3	60	4.51	4.34	3.09	2.97	10.42	10.36	1.43	1.37
11	50	3	80	3.78	3.78	2.26	2.40	9.50	9.59	1.10	1.09
12	50	3	60	4.46	4.34	3.01	2.97	10.48	10.36	1.45	1.37
13	50	3	60	4.48	4.34	3.12	2.97	10.41	10.36	1.43	1.37
14	25	4	70	3.15	3.15	2.16	2.19	9.60	9.62	1.00	1.10
15	50	3	60	4.51	4.34	2.95	2.97	10.2	10.36	1.45	1.37
16	50	3	40	3.85	3.83	2.48	2.45	9.60	9.89	0.92	0.93
17	25	2	70	3.25	3.21	2.15	2.16	9.36	9.4	1.12	1.29
18	50	1	60	2.87	3.14	2.03	2.13	9.80	9.64	1.32	1.36
19	50	5	60	2.90	2.98	2.50	2.49	9.90	9.8	0.86	0.85
20	25	4	50	2.96	2.95	2.38	2.17	9.53	9.6	0.75	0.74

X_1: Ethanol concentration (%); X_2: time (h); X_3: temperature (^{0}C); (Y_1): total phenolic content (mgGAE/g) (TPC); (Y_2): total flavonoid content (mgCAE/g) (TFC); (Y_3): DPPH-radical scavenging activity (% inhibition); (Y_4): cupric reducing antioxidant capacity (µM ascorbic acid equivalent) (CUPRAC).

Table S2: Independent process variables with experimental ranges and levels for heat reflux extraction of ripe Ajwa dates pulp (RADP).

**Input variables**	**Variable range and levels (coded)**						
	**unit**	**Code**	**-α**	**-1**	**0**	**+1**	**+α**
Ethanol concentration (EtOH)	%	X_1	0	25	50	75	100
Time	h	X_2	1	2	3	4	5
Temperature	^{0}C	X_3	40	50	60	70	80

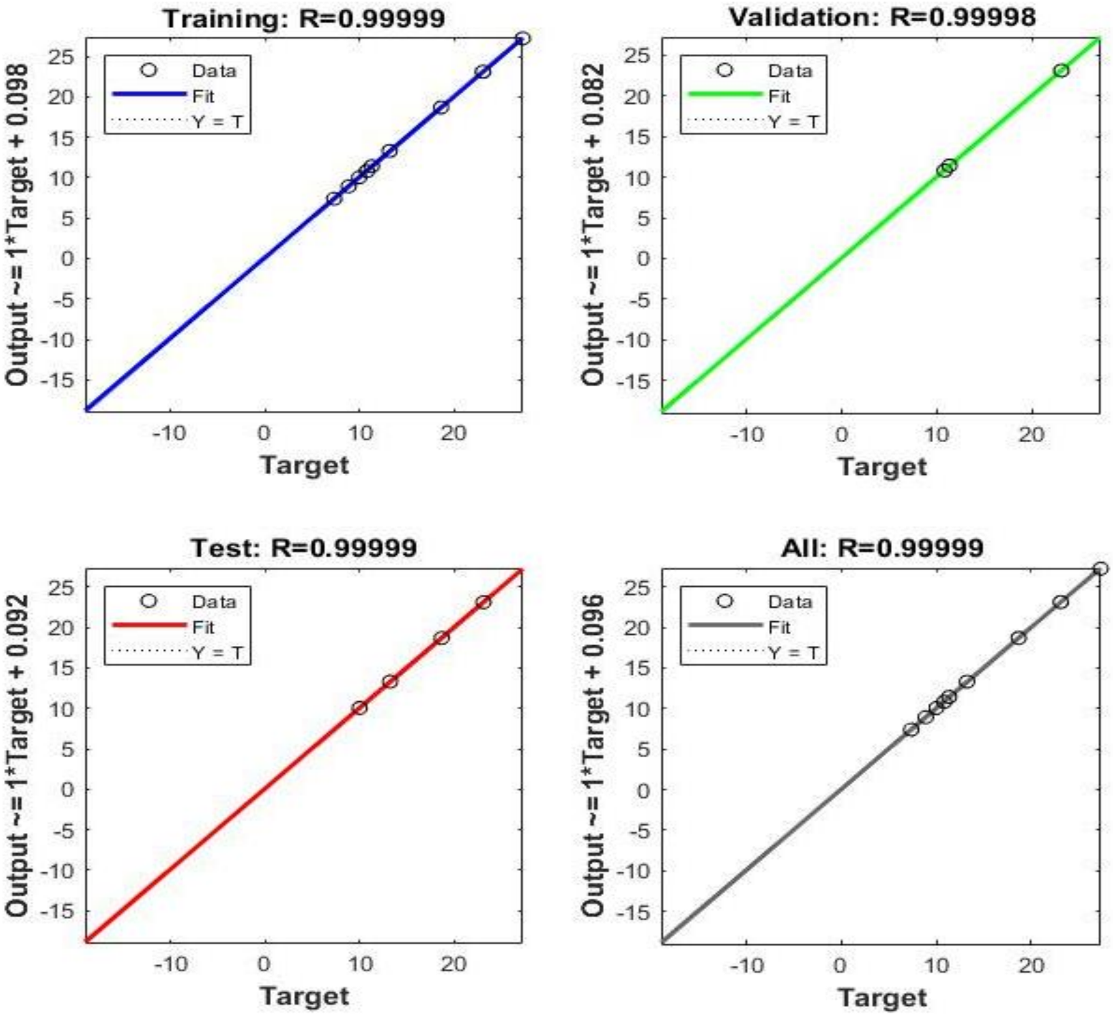

Figure S1: The R^2 value of training, validation, test and overall for TPC during ANN.

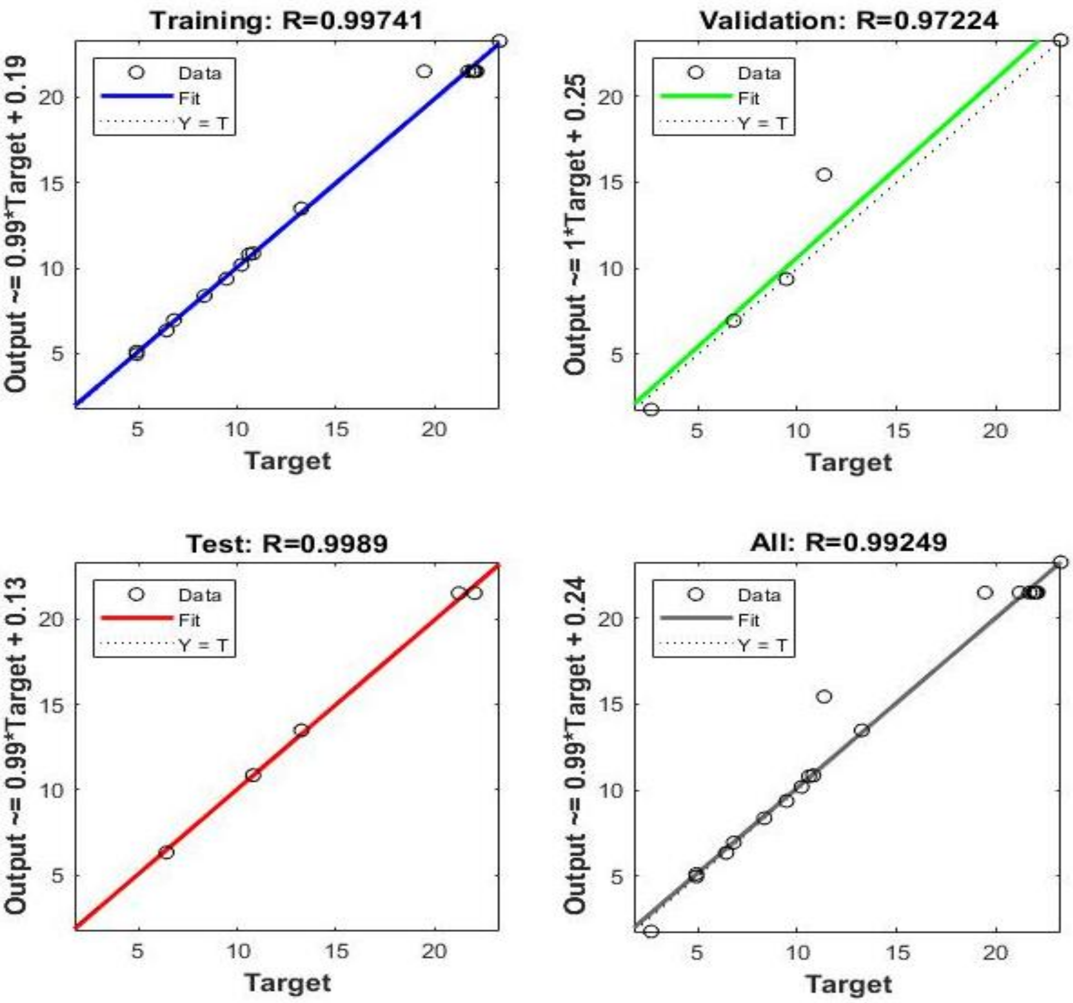

Figure S2: The R^2 value of training, validation, test and overall for TFC during ANN.

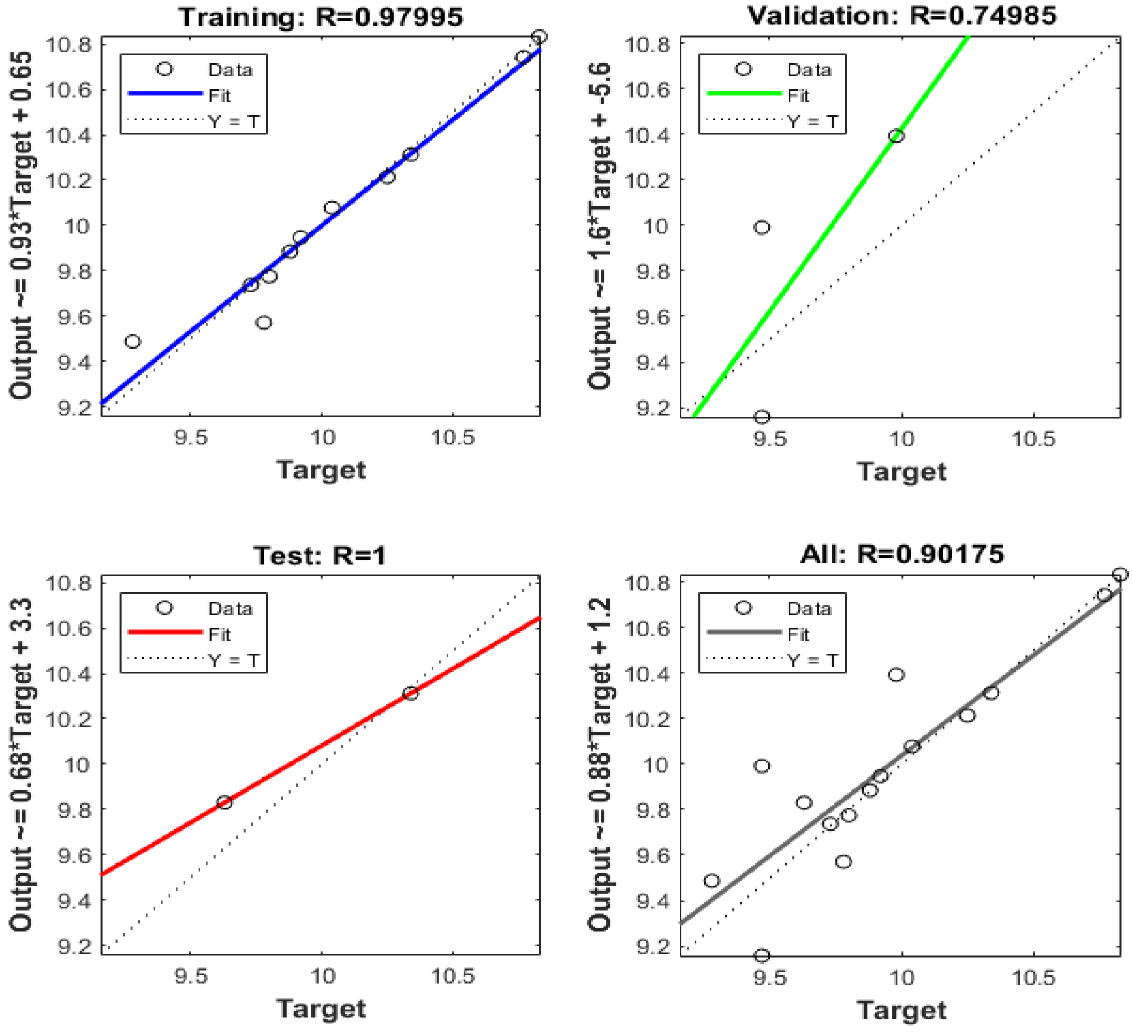

Figure S3: The R^2 value of training, validation, test and overall for DPPH during ANN.

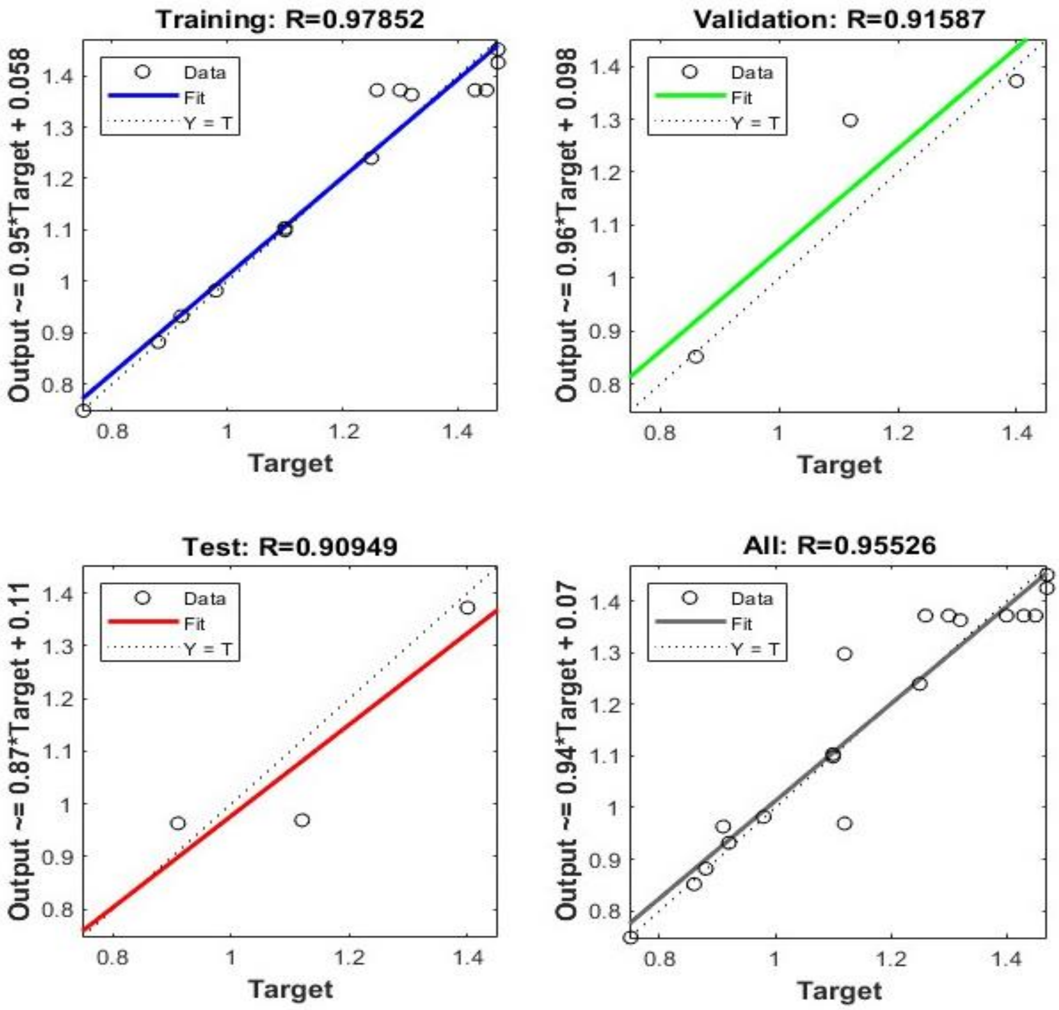

Figure S4: The R^2 value of training, validation, test and overall for CUPRAC during ANN.

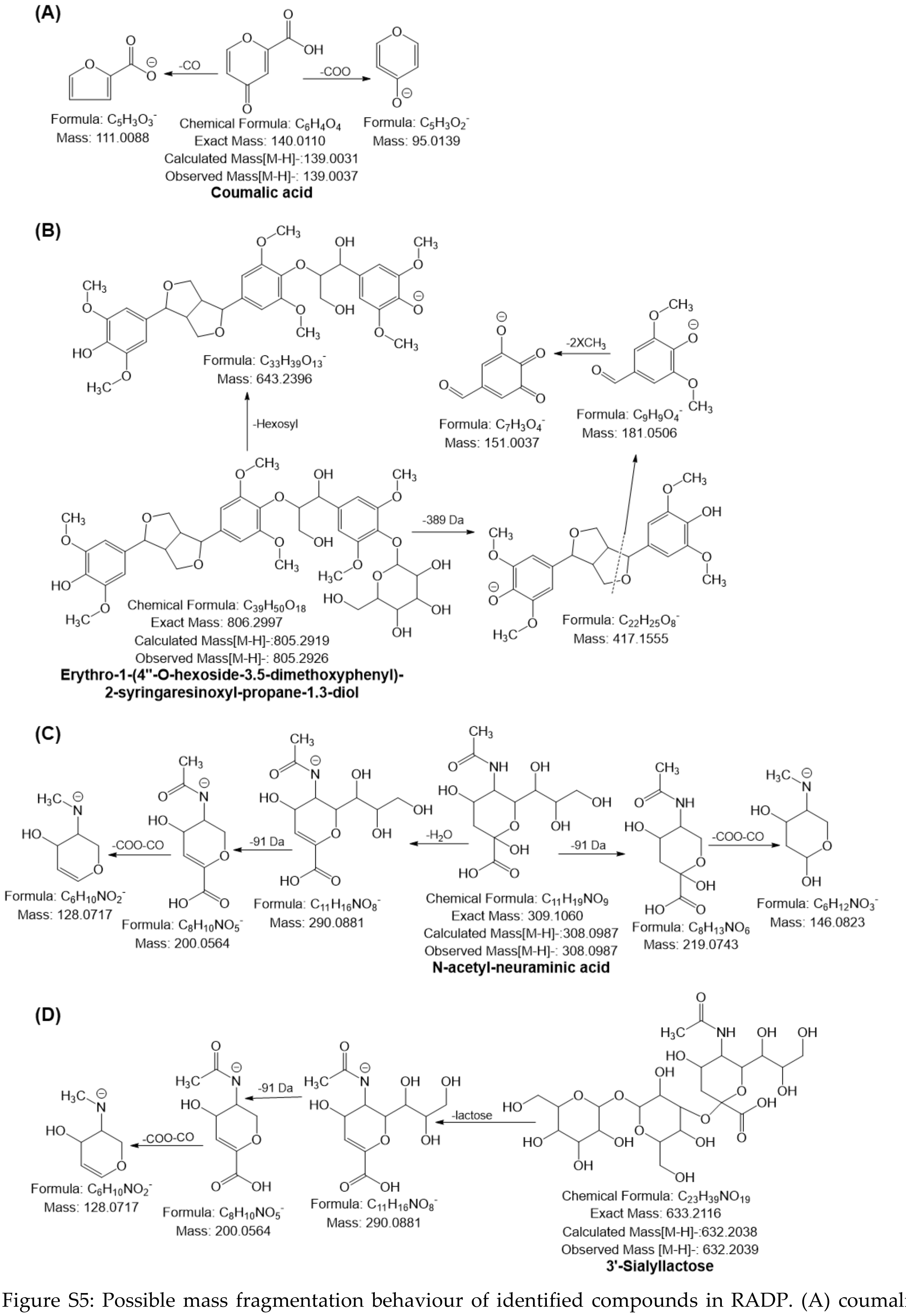

Figure S5: Possible mass fragmentation behaviour of identified compounds in RADP. (A) coumalic acid, (B) Erythro-1-(4''-O-β-D-glucopyranoside-3,5-dimethyoxyphenyl)-2-syringaresinoxyl-propane-1,3-diol, (C) N-acetyl-α-neuraminic acid, and (D) 6′-sialyllactose.

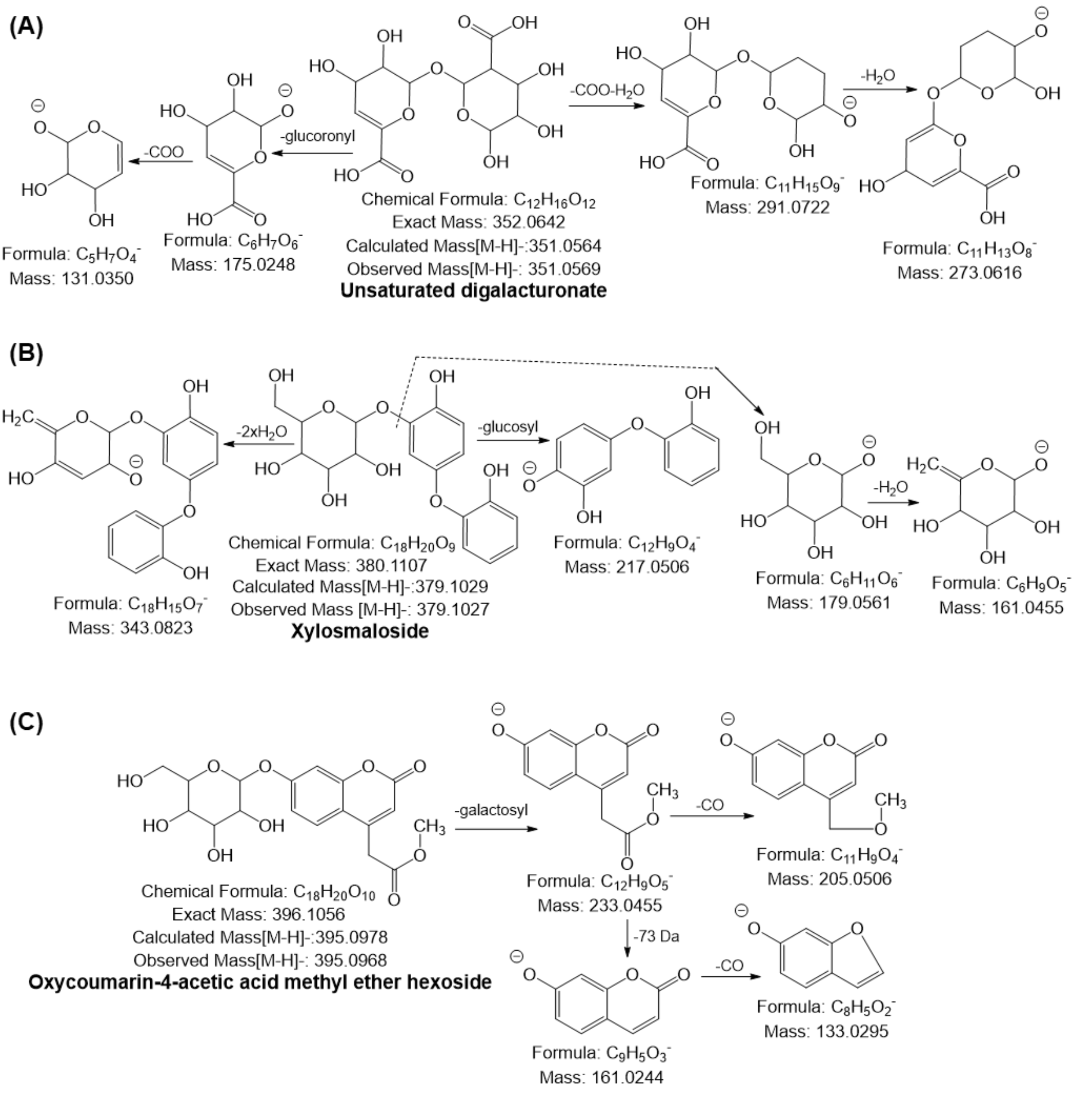

Figure S6: Possible mass fragmentation behaviour of identified compounds in RADP. (A) Unsaturated digalacturonate, (B) Xylosmaloside and (C) Oxycoumarin-4-acetic acid methyl ester hexoside.
